# Dumbbell configuration of silicon adatom defects on silicene nanoribbons

**DOI:** 10.1038/s41598-021-93465-5

**Published:** 2021-07-13

**Authors:** Huynh Anh Huy, Quoc Duy Ho, Truong Quoc Tuan, Ong Kim Le, Nguyen Le Hoai Phuong

**Affiliations:** 1 Department of Physics, School of Education, Can Tho University, Can Tho City, Viet Nam; 2 Faculty of General Sciences, Can Tho University of Technology, Can Tho City, Viet Nam; 3 Department of Physics, College of Natural Sciences, Can Tho University, Can Tho City, Viet Nam; 4 Faculty of Basic, Tay Do University, Can Tho city, Viet Nam

**Keywords:** Electronic properties and materials, Electronic structure

## Abstract

Using density functional theory (DFT), we performed theoretical investigation on structural, energetic, electronic, and magnetic properties of pure armchair silicene nanoribbons with edges terminated with hydrogen atoms (ASiNRs:H), and the absorptions of silicon (Si) atom(s) on the top of ASiNRs:H. The calculated results show that Si atoms prefer to adsorb on the top site of ASiNRs:H and form the single- and/or di-adatom defects depending on the numbers. Si absorption defect(s) change electronic and magnetic properties of ASiNRs:H. Depending on the adsorption site the band gap of ASiNRs:H can be larger or smaller. The largest band gap of 1 Si atom adsorption is 0.64 eV at site 3, the adsorption of 2 Si atoms has the largest band gap of 0.44 eV at site 1-D, while the adsorption at sites5 and 1-E turn into metallic. The formation energies of Si adsorption show that adatom defects in ASiNRs:H are more preferable than pure ASiNRs:H with silicon atom(s). 1 Si adsorption prefers to be added on the top site of a Si atom and form a single-adatom defect, while Si di-adatom defect has lower formation energy than the single-adatom and the most energetically favorable adsorption is at site 1-F. Si adsorption atoms break spin-degeneracy of ASiNRs:H lead to di-adatom defect at site 1-G has the highest spin moment. Our results suggest new ways to engineer the band gap and magnetic properties silicene materials.

## Introduction

Since the first reparation in 2004, graphene has gained tremendous attention due to its attractive properties^1,2^. However, potential applications of graphene have been limited because of its zero band gap^3^. The existence of graphene inspired the study of other single-atom-layer materials that show promising characteristics^4,5^. Among those potential materials is silicon single-atom-layer that is later named as silicene. Silicence that is a hexagonal mesh of silicon atoms was first studied by Takeda and Shiraishi^6^. It gains a lot of attention because it has comparable electronic characteristics as graphene i.e. the Dirac cone and the quantum spin Hall effect behaviors^4,7,8^ and it can be integrated into semiconductors devices much more easier than graphene by using mature semiconductor techniques. Compare to the flat structure of graphene, silicene has a buckled structure with one of two silicene sublattices shifts in the direction perpendicular to the atomic plane, it allows the band gap of silicene can be manipulated easily by applying an external filed^6^. Ciraci group^9^ found the buckled height of silicene is 0.44 Å by using ab-initio molecular dynamics simulations on the basis of density functional theory. Even though the first theoretical study of silicene was reported in 1994^6^, the first experimentally successful synthesis large-area silicene on Ag (111) was only reported in 2012^10–12^, and the first application of silicene was only introduced in 2015 by Tao et al*.*^13^*,* silicene FETs with the carrier mobility of devices around 100 cm^2^v^−1^ s^−1^. The carrier is less than expected, but it can be increased by tuning the band structure and enhancing the charge transportation of silicene^13^.

Before the experimental preparation of pristine silicene on Ag (111) substrate, nanoribbons form of silicene has been fabricated on Ag(001)^14^ and Ag(110)^15–17^. The width of silicene nanoribbons (SiNRs) on Ag was measured at 1.6 nm^15^. The sp^2^ hybridization electronic configuration and graphene-like band dispersion on the Ag(110), supported SiNRs were then reported by De Padova et al.^16,17^. There are two types of silicene nanoribbons, zigzag (ZSiNRs) and armchair (ASiNRs), it depends on structural edges. ZSiNRs that terminated with hydrogen atoms is a zero-gap semimetal^18^. ASiNRs with hydrogen atoms on the edges open a gap between the valence band maximum (VBM) and the conduction band minimum (CBM), the width of gap depends on the width of the ribbon^19^.

Defects in a material are almost unavoidable in the fabrication and processing, and sometimes, they are introduced through the synthesize procedure purposively for controlling some specific applications^20,21^. Besides vacancy, extended line defects, defects form by the adsorption on the surface are often studied in group IV single layer materials like graphene and silicene by using density functional theory^22–24^. In pure graphene and H terminated graphene, carbon (C’—we use the same notation as in ref^23^) is more favorable at the bridge site between 2 C atoms^23^, while silicon is reported to be more energetically on a top site of Si to form adatom defect(s)^22^. Even though, the absorption of C and Si on the top of graphene and silicene, respectively, has shown the widen of the band gap and the spin polarization variation, there is no report of Si absorption on the surface of ASiNRs:H. Controlling electronic and magnetic properties of silicene material is one of interested topic that attracts a lot of researchers. In this paper, we will study ASiNRs:H with silicon (Si) atom(s) adsorbed on the surface of ASiNRs:H aiming for engineering the band gap and magnetic properties of silicene materials. The next part of the report is computational calculation details followed by results and discussion. The final part is the conclusion.

## Computational details

**Figure 1:**
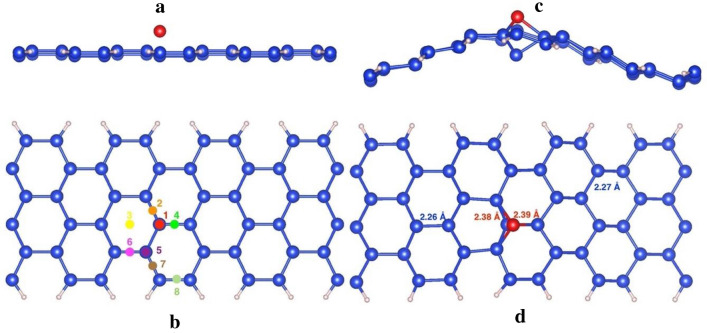
ASiNRs:H from the side (**a**) and top view with 8 different absorbed positions (**b**). ASiNRs:H with a single—diatom (site 1) from the side (**c**) and top view (**d**). The blue and pink balls represent for Si and H in ASiNRs:H, respectively. The red ball is the adsorbed Si’ atom at site 1.

**Figure 2:**
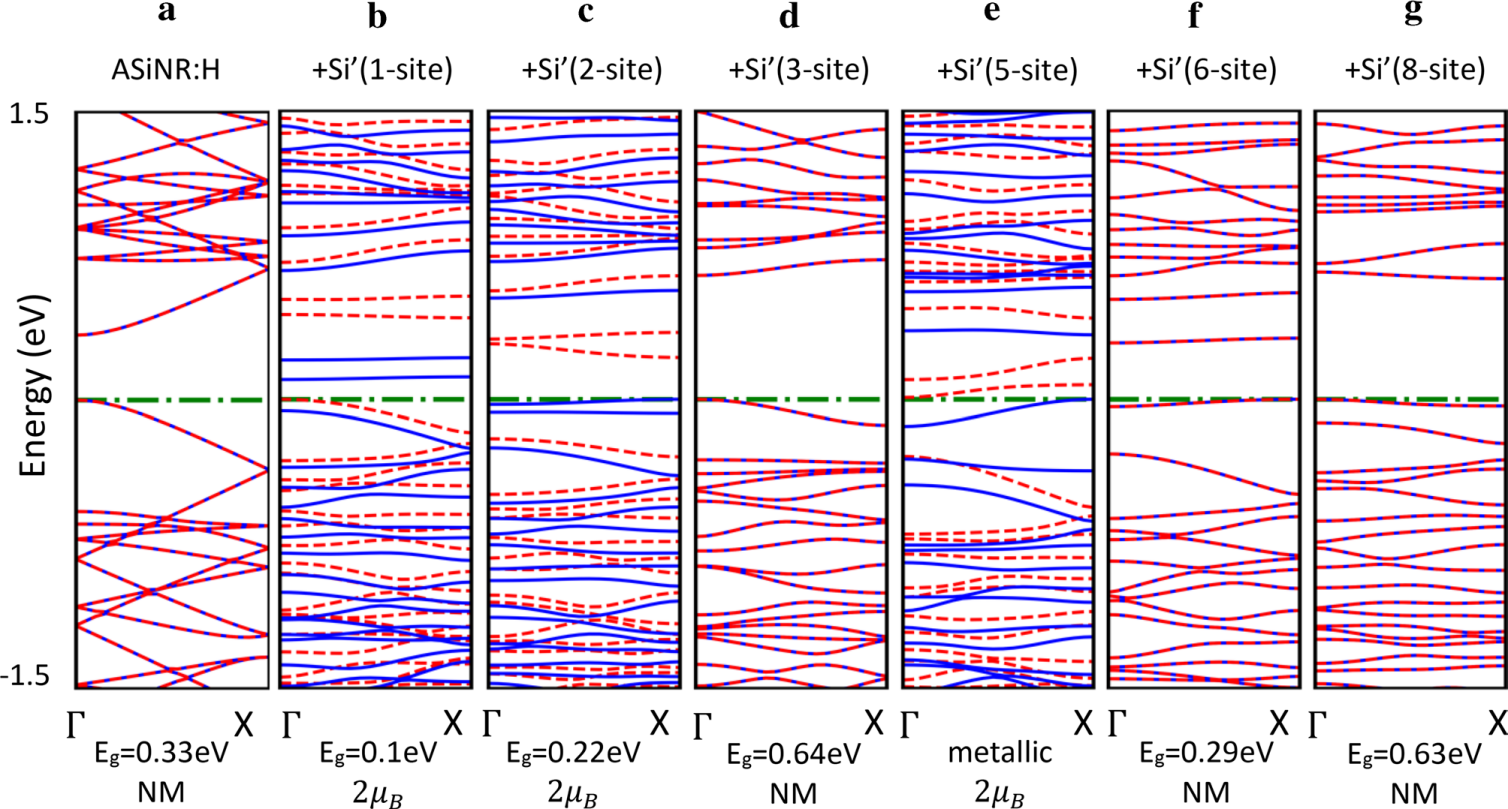
Effect of Si’ adsorption on the electronic and magnetic properties of ASiNRs:H. (**a**) Pure hydrogen saturated armchair nanoribbon, between (**b**) and (**g**) are the same nanoribbon with Si’ atom adsorbed on the top of site 1, 2, 3, 5, 6, and 8. The Fermi-level is set a zero. The dashed red and solid blue lines are spin-up and spin-down bands, respectively. E_g_ and NM stand for the band gap and nonmagnetic, respectively.

**Figure 3:**
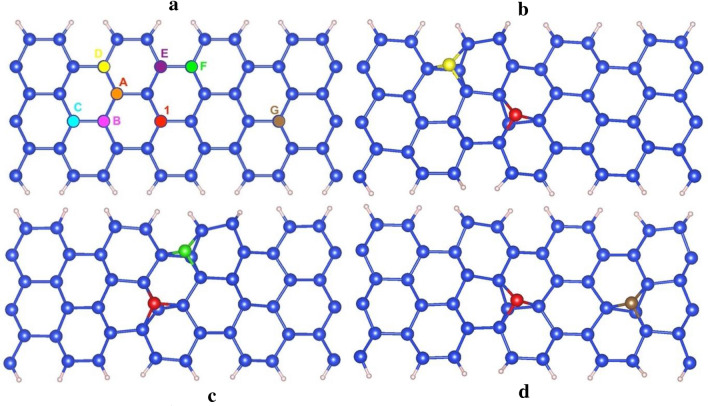
ASiNRs:H from the top view with 1 Si’ atom adsorb at site 1 and another Si’ atm at different positions from A to G (**a**). ASiNRs:H with di-adatom at site 1-D (**b**), site 1-F (**c**), and site 1-G (**d**). The blue and pink balls represent for Si and H in ASiNRs:H, respectively. The red, yellow, green, and brown balls are the adsorbed Si’ atom at site 1, D, F, and G, respectively.

**Figure 4:**
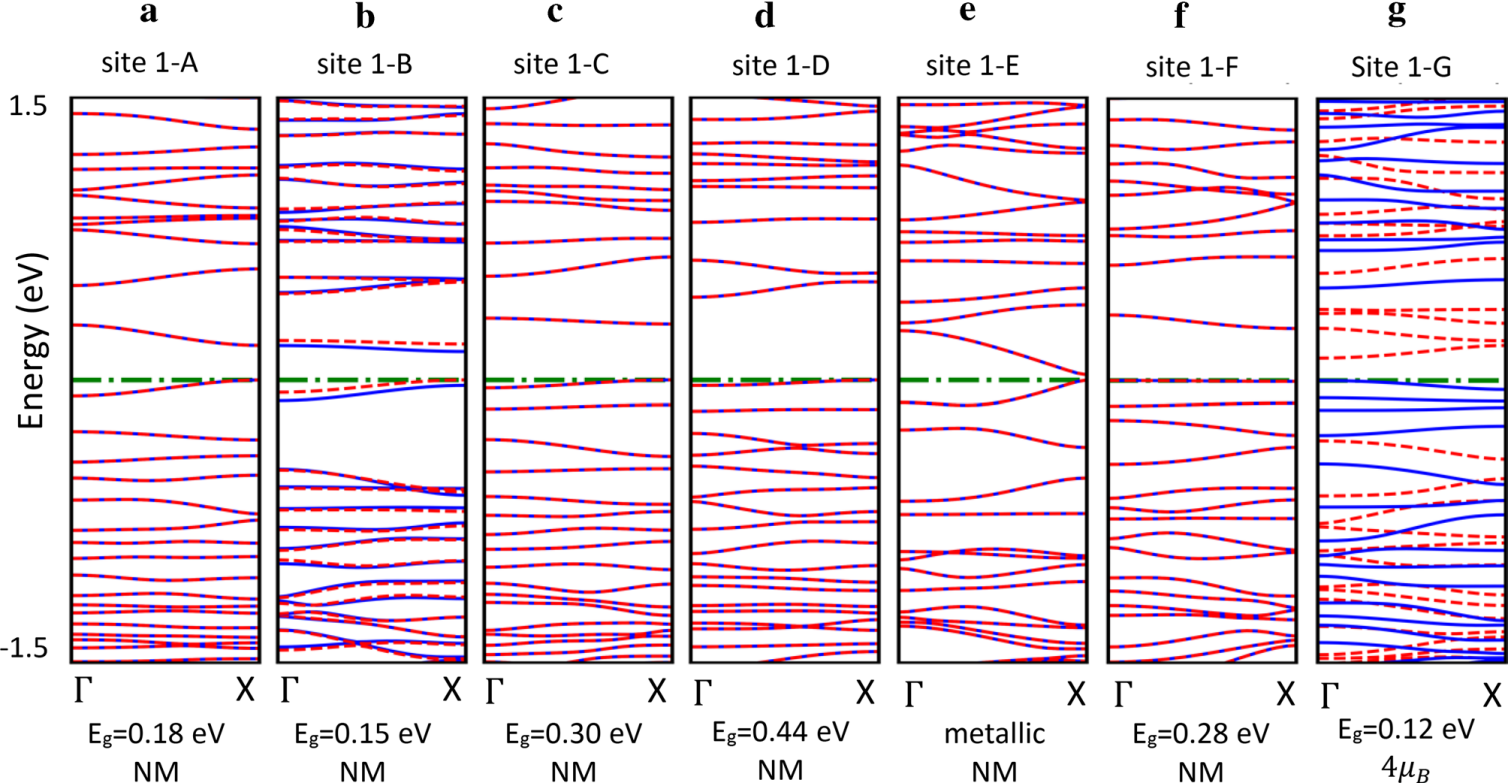
Effect of 2 Si’ atoms adsorption ASiNRs:H. From (**a**) to (**g**) are electronic structure and magnetic properties of ASiNRs:H with di-adatom defect from site 1-A to 1-F, E_g_ and NM stand for the band gap and nonmagnetic, respectively.

In the present calculations, we performed with density functional theory used the Vienna Ab-initio Simulation Package (VASP 5.4.4), the projector augmented wave method (PAW)^25^, and the Generalized Gradient Approximation (GGA) or Perdew, Burke, and Ernzerh (PBE)^26^ for exchange and correlation. The plane-wave basis set was truncated at 420 eV and the kinetic energy cut-off for the augmentation charges was 840 eV. The ASiNRs:H and ASiNRs:H adatom(s) defect band structures calculations were calculated in supercells (64 atoms for pure ASiNRs:H) with a vacuum space of around 15 Å to avoid the interaction between the nanoribbon and its periodic images. The convergence condition of 10^–4^ eV was applied for the self-consistent electronic energy and the relaxation of all ions was carried out until the force on each ion was smaller than 0.02 eV/Å. The structure optimizations were carried out with a 1 × 1 × 3 approximations for the Brillouin zone sampling, where 3 is only applied for the periodic direction. Convergence of the k-point sampling was checked again by applying a 1 × 1 × 5 set. For plotting band structure in Figs. 2 and 4, Pymatgen^27^ python-based code was used. VESTA3^28^ was used to display the balls and stick structure models of ASiNRs:H in Figs. 1 and 3.

## Results and discussions

We study ASiNR:H with the width of 6 silicon atoms, Fig. 1a shows the top view and side view of 6-ASiNR:H geometry structures, those edges are terminated with hydrogen (H) atoms. Blue and cyan balls signify Si and H atoms. ASiNR:H has buckle structure with bonding between Si atoms are slightly smaller than 2.23 Å, bonding between Si and H atoms is 1.49 Å.

For studying the stability of Si atom(s) on the surface of ASiNRs:H, we calculate formation energy as follow: *E*_ad_ = *E*_def_ – (*E*_ASiNRs:H_ + *n*.*E*_Si_) Where: *E*_def_ is the total energy of Si diatom ASiNRs:H, *E*_ASiNRs:H_ is the total energy of ASiNRs:H, *n* is the number of adsorbed Si atoms, *E*_Si_ is the energy of an isolated silicon atom.

We study the adsorption of Si’ atoms (Si’ will be used for silicon atoms that adsorb on the top of ASiNRs:H from now on) on the surface of ASiNRs:H by placing a Si’ atom on top of ASiNRs:H. Because the nanoribbons break Si_6_ rotation symmetry, we place Si’ atom at 8 different positions as in Fig. 1b. The Si’ atom is placed on top (T, top of an Si atom – site 1 and 5), bridge (B, above the Si–Si bond, site 2, 4, 6, 7 and 8) and hollow (H, above the center of hexagons, site 3) sites.

Table 1 shows calculated formation energies of Si’ on the surface of ASiNRs:H. Our calculation found that Si’ refers to be absorbed on the top of a center top site of ASiNRs:H, site 1 (Fig. 1c,d). The absorption on the top of a Si site forms a so-called “dumbbell configuration”^29,30^, the dumbbell configuration was also found the be the most favorite absorbed configuration in pure silicene^22^. When the dumbbell configuration is formed (Fig. 1c,d), the bondings between the adatom and neighbor Si atoms increase to around 2.38 Å. The distances between Si away from the adatom also rise to 2.27 Å. That could be due to the fact that the adatom move out of the plane and the others move forward the left position. The distances from Si to H atoms after the adsorption is the same as before. After the Si atom is absorbed, ASiNR:H structure has a concave form with the peak at the absorbed site (the red Si ball, in Fig. 1c).

**Table 1:** The adsorption energy of 2 Si’ atoms on ASiNRs:H surface.

Absorbed site	1	2	3	4	5	6	7	8
Absorption energy (eV)	− 2.13	− 1.71	− 1.69	− 1.95	− 1.78	− 2.03	− 1.74	− 1.99

Figure 2 shows band structures and magnetic properties of pure ASiNRs:H as well as ASiNRs:H with Si’ absorbed at different sites. As can be seen from the Fig. 2 that the pure ASiNRs:H is a nonmagnetic (NM) semiconductor. However, when a Si’ atom absorbs on the surface, it can turn into a metallic or a larger band gap semiconductor with NM or spin-polarization, depending on the absorbed site. More specifically, when Si’ is added to site 1, 2 and 5, spin degeneracy is broken, the energy of spin-down is pushed down and lower than the spin-up. While the absorptions at site 1 and 2 only narrow the band gap of ASiNRs:H. The site 5 adatom defect turns ASiNRs:H into metallic. The absorption of Si’ at the other sites does not change the NM properties of ASiNRs:H. The defect at site 3 and 8 widen the band gap up to almosr 0.65 eV. It should be noted here that DFT-GGA is well-known for band-gap underestimation, for examination the correct band gap, a high computational cost method should be employed like HSE or GW.

As discussed above, the absorption of Si’ at 1-site is the most favorable, however site 1 adatom defect closes the band gap to 0.1 eV that is smaller than bare ASiNRs:H. The absorption of Si on ASiNRs:H should be avoided in the case of aiming a wider band gap of silicene. But the absorption of a silicon atom can be considered for making magnetic devices. With implantation technology Si’ atom can be put at the desire site, in that situation a wlarger band gap ASiNRs:H can be made with the adsorption at site 3 or 8.

We also study the possibility of a second silicon atom absorption on the surface of ASiNRs:H. Figure 3 shows the absorption of 2 Si’ atoms at 2 site. The first added Si’ atoms is placed at site 1 that has the lowest absorption energy as discussed above, terefore we use the same name with number for this site. The second Si’ is added in 7 other sites marked from A to G as in the Fig. 3. The adsorption of 2 Si’ atoms on ASiNRs:H form di-adatom structures, that is similar to the adsorption of 2 Si’ atoms in pure silicene^22^. The formation energies of di-adatoms are shown in Table 2.

**Table 2:** The adsorption energy of 2 Si’ atoms on ASiNRs:H surface.

Absorbed sites	1-A	1-B	1-C	1-D	1-E	1-F	1-G
Absorption energy (eV)	− 1.99	− 1.39	− 1.34	− 2.20	− 2.25	− 2.51	− 1.55

Figure 4 indicate electronic strucutures of Si di-adatom defects with adsorbed positions from site 1-A to 1-D. The absorption of 2 Si’ atoms on the top of ASiNRs:H causes a significant reduced band gap at site 1-A (0.18 eV, Fig. 4a) and quite similar band gap at site 1-B (0.12 eV, Fig. 4b). The largest band gap of a di-adatom defect ASiNRs:H is an indirect one (0.44 eV, Fig. 4d) with the absorption at site 1-D. However, the formation energy of site 1-D di-adatom is higher than at site 1-F (− 2.20 eV vs − 2.51 eV). 1-F di-adatom has the band gap of 0.28 eV (Fig. 4f) that is a little bit smaller than the most favorable single-adatom defect (at site 1). The Fig. 1e show that the 1-E di-adatom is a metallic. We also examine spin-polarization of di-adatom defect, while a further absorption of a Si’ atom from site A to F turn single-adatom defect to nonmagnetic, the defect at site 1-G (Fig. 1g) has $4{\mathrm{\mu }}_{\mathrm{B}}$ spin moment that is the highest polarization even compare to single Si’ adsorption.

The formation energies of di-adatom defect (Table 2) have shown that di-adatom defect in ASiNRs:H (site 1-F) is more favorable that the pristine and single-adatom ASiNRs:H. The di-adatom defect (site 1-F) narrow the band gap of ASiNRs:H from 0.33 eV to 0.28 eV, therefore the defect should be avoided in the case looking for a larger band gap of ASiNRs:H. The di-adatom defect (site 1-F) turn the spin-polarization ($2{\mu }_{B}$) (site 1) into no magnetization, while the adsorption at site 1-G increases the spin-polarization to $4{\mu }_{B}$. Even though the formation energy of 1-G di-adatom defect has higher formation energy than 1-F di-adatom, that defect can be procured by implantation technique in experiment. Thus, the 1-G di-adatom defect should be considered carefully for magnetic devices.

## Conclusion

In summary, by using density functional theory with PBE exchange–correlation, we have studied pure ASiNRs:H and the adsorption of 1 and 2 Si’ atoms ASiNRs:H surface. The calculated adsorption energies show that the absorption of 2 Si’ atoms on ASiNRs:H surface is more stable than the adsorption of 1 Si’ atom and pure ASiNRs:H with single Si atom(s). The Si’ atoms prefer to adsorb on the top of Si sites in ASiNRs:H and form the single- or di-adatom defects. The adatoms defect change the band gap of ASiNRs:H. Depending on the adsorbed sites the band gap can be larger or smaller, the site 3 and 8 single Si’ atom adsorption have the largest band gap at around 0.65 eV, while the adsorption at site 5 and 1-E di-adatom defect are metallic. The magnetic properties of ASiNRs:H can also be tuned by adsorption with the adsorption of Si atoms, the 1-G di-adatom defect has the highest spin moment at $4{\mu }_{B}$.
